# Advances in subpopulation separation and detection of extracellular vesicles: for liquid biopsy and downstream research

**DOI:** 10.7150/thno.106459

**Published:** 2025-01-01

**Authors:** Zi-Xiu Liu, Gang Chen, Zi-Li Yu

**Affiliations:** 1 State Key Laboratory of Oral & Maxillofacial Reconstruction and Regeneration, Key Laboratory of Oral Biomedicine Ministry of Education, Hubei Key Laboratory of Stomatology, School & Hospital of Stomatology, Wuhan University, Wuhan, China.; 2 Department of Oral and Maxillofacial Surgery, School and Hospital of Stomatology, Wuhan University, Wuhan, China.; 3 TaiKang Center for Life and Medical Sciences, Wuhan University, Wuhan, China.; 4 Frontier Science Center for Immunology and Metabolism, Wuhan University, Wuhan, China.

**Keywords:** extracellular vesicle, subpopulation, separation, detection, liquid biopsy

## Abstract

Extracellular vesicles (EVs) are carriers of a diverse array of bioactive molecules, making them valuable clinical tools for liquid biopsy in disease diagnosis and prognosis evaluation. These molecules play critical roles in various physiological and pathological conditions, and effective separation of EVs is essential to achieve these objectives. Due to the high heterogeneity of EVs, particularly with regard to their cargo molecules, merely isolating the general EV population is inadequate for liquid biopsy and biological function studies. Therefore, separating EV subpopulations becomes crucial. Traditional separation methods, such as differential ultracentrifugation and size exclusion chromatography, along with burgeoning techniques like classical microfluidic chips and covalent chemistry, often prove time-consuming, yield low purity, and have limited ability to address cargo heterogeneity. Thus, precise separation of EV subpopulations is of utmost importance. Additionally, detecting subpopulation-specific cargo is vital for validating the effectiveness of separation methods and supporting clinical biopsy applications. However, reviews that focus specifically on detection methods for EV subpopulations are limited. This paper provides a comprehensive overview of the methods for separating and detecting EV subpopulations with surface marker heterogeneity, comparing the advantages and limitations of each technique. Furthermore, it discusses challenges and future prospects for these methods in the context of liquid biopsy and downstream research. Collectively, this review aims to offer innovative insights into the separation and detection of EV subpopulations, guiding researchers to avoid common pitfalls and refine their investigative approaches.

## Introduction

Extracellular vesicles (EVs) are nanoscale membrane-bound structures secreted by cells 1. Almost all cell types are capable of secreting EVs 2. As crucial carriers of intercellular communication, EVs encapsulate a diverse array of bioactive molecules from their parent cells, and can transfer these molecules to recipient cells, thereby influencing their biological functions and participating in various physiological and pathological processes 3-6. Since EVs carry information from their parent cells, they can reflect the type and state of these cells, which forms the theoretical basis for disease liquid biopsy using EVs 7. Due to their rich content of biomolecules, resistance to enzymatic degradation, and wide distribution in body fluids, EVs have emerged as highly promising biomarkers for disease liquid biopsy, playing a key role in the early diagnosis and prognostic monitoring of various diseases, including malignant tumors 8-12.

However, growing clinical evidence suggests that, traditional liquid biopsy methods for obtaining and detecting total EVs, such as tumor-derived EVs, face limitations in detection accuracy. This limitation largely arises from the high heterogeneity of EVs, which includes not only size differences but also significant variability in their molecular cargos 10,13,14. For example, the same cell type can secrete EVs with different molecular compositions under various physiological and pathological conditions. Furthermore, EVs from different cell types may carry some identical bioactive molecules. Numerous experimental studies have demonstrated that, even within a single EV population, there can be considerable variation in both characteristics and biological functions 15-17. Therefore, subclassifying EVs based on their molecular cargo is of great importance. By categorizing EV populations into subpopulations and conducting fine analyses, it is possible to address the challenges posed by EV heterogeneity more effectively. This approach not only aids in elucidating the roles of different EV subpopulations in biological activities, but also has the potential to enhance the efficiency and accuracy of liquid biopsies 18,19.

**Figure 1:**
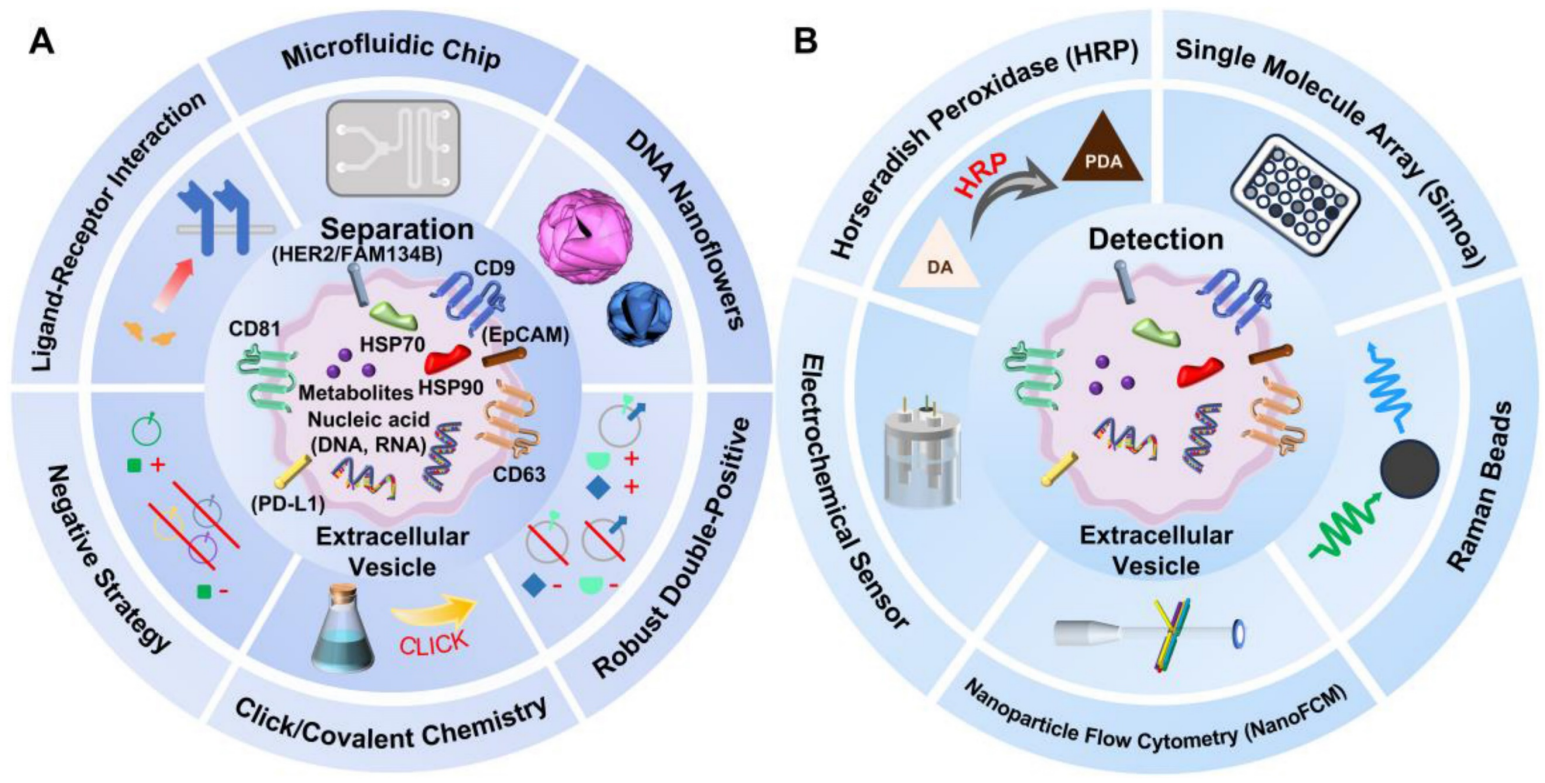
(A) Classical and some potential cargo molecules carried by EVs, along with separation methods for EV subpopulations, including approaches based on microfluidic chip, click/covalent chemistry, DNA nanoflowers, robust double-positive strategy, receptor-ligand interaction and negative strategy. (B) Detection methods for EV subpopulations, including approaches based on NanoFCM, Simoa, HRP, electrochemical sensor and Raman beads.

Although traditional and some burgeoning EV separation methods based on particle size, density, or specific physical properties of EVs in electrical, acoustic, or fluidic environments can address size heterogeneity to some extent, they still struggle with the fine subpopulation separation based on the complex molecular cargo of EVs. Moreover, reviews on existing methods for EV subpopulation detection are relatively scarce. Therefore, this review aims to provide a detailed summary of recent advances in the separation and detection of EV subpopulations, and to compare the advantages and limitations of various methods (**Figure 1A-B**).

## EV subpopulations: key players in intercellular communication and potential biomarkers for liquid biopsy

EVs are nanoscale vesicles secreted by nearly all cell types and are widely present in body fluids 1,2. Historically, EVs were considered “waste” products of cell maturation, used to remove molecular byproducts from cells, leading to their functions being long underestimated 20,21. However, EVs can carry various cargo molecules, including proteins, nucleic acids, and metabolites, which they transfer from parent cells to recipient cells 3. As a result, EVs play precise roles in intercellular communication and epigenetic regulation in both normal physiological and pathological states, such as immune responses, inflammation, and cancer 22-26. Studies have shown that EVs derived from immune cells can present antigens and initiate immune responses 27. Our previous research has also confirmed that, EVs derived from tumor cells carry programmed death ligand 1 (PD-L1) on their surfaces, which can directly bind to programmed cell death protein 1 on CD8^+^ T cells, thereby mediating systemic immune suppression at sites distant from the primary tumor 28,29.

Moreover, because EVs from cancerous and healthy tissues exhibit significant differences in expression levels and characteristic molecules, they are considered potential biomarkers for clinical diagnosis and prognosis 11,12,30,31. By analyzing the expression of specific cargo molecules carried by EVs separated from body fluids such as blood, urine, and saliva, it is possible to facilitate early disease diagnosis and prognostic monitoring. Many studies have focused on the protein, RNA, and other cargo molecules carried by EVs for clinical liquid biopsy applications 14. For instance, elevated expression levels of PD-L1 and epithelial cell adhesion molecule (EpCAM) are commonly associated with various types of cancer 32. More specifically, human epidermal growth factor receptor 2 (HER2) is linked to breast cancer, alpha-fetoprotein to hepatocellular carcinoma, and the increased expression of epidermal growth factor receptor (EGFR) on EVs correlates with a higher risk of non-small cell lung cancer 32. Beyond cancer, EVs also serve as biomarkers in other clinical liquid biopsies, such as *α*-synuclein for Parkinson's disease 33,34, Wilms' tumor 1 transcription factor for podocyte injury 35,36, and asialoglycoprotein receptor 1 for liver disease 37,38. Compared to traditional methods, the detection of characteristic molecules carried by EVs in body fluids can provide faster results, facilitating more timely clinical decision-making. Furthermore, through design and modification, EVs can be engineered to deliver therapeutic cargos, offering potential for drug delivery and disease treatment 39,40.

However, EVs exhibit high heterogeneity 10,13,14. Based on their origin, EVs are primarily classified into exosomes, microvesicles, and apoptotic bodies, with increasing size and differing physical properties, such as density 41,42. The term “exosome” refers to EVs derived from internal compartments of the cell, which are released via the multivesicular body, whereas “ectosome” refers to EVs originating from the cell surface. However, according to the Minimum Information for Studies of Extracellular Vesicles 2023 43, terms based on presumed biogenesis pathways should be used with caution and only when there is strong supporting evidence, unless the EV population has been specifically separated and characterized. This caution is due to the lack of universal molecular markers for biogenesis-based EV subtypes 43. The present recommended terminology emphasizes characteristic differences such as size, density, molecular composition, or cellular origin 43. For example, small EVs (sEVs) are typically described as <200 nm in diameter, while large EVs are >200 nm. These terms are encouraged for use. Notably, sEVs include both small ectosomes and exosomes, and the terms are not synonymous 43. Despite these updated guidelines, many researchers still fail to adhere to these principles, which can lead to confusion.

As mentioned above, EVs not only exhibit heterogeneity in their physical properties but also show considerable diversity in the molecules they carry. Our previous research indicates that tumor cells can secrete both PD-L1^+^ and PD-L1^-^ EVs 28. Furthermore, PD-L1^+^ EVs are not exclusively derived from tumor cells; immune cells can also secrete such EVs 17. This suggests that the same cell type can secrete different EV subpopulations under various states, and different cells may secrete similar subpopulations of EVs. The heterogeneity of cargo molecules leads to significant diversity among EV subpopulations, presenting both opportunities and challenges. In clinical liquid biopsy, accurately separating and detecting specific EV subpopulations can aid in precise diagnosis and prognosis, and in devising targeted treatment plans. However, focusing solely on a single indicator for EV subpopulation separation and detection may lead to errors and biases in prognosis. For instance, while patients with high levels of PD-L1^+^ EVs are generally considered less likely to benefit from immunotherapy, some patients with high levels of PD-L1^+^ EV can still respond well to immunotherapy 44. This discrepancy arises because some PD-L1^+^ EVs are secreted by immune cells and co-express other molecules, resulting in weaker immunosuppressive functions compared to those secreted by tumor cells. Therefore, the molecular heterogeneity of EVs leads to variations in their biological functions. Subdividing EVs based on cargo heterogeneity and detecting these subpopulations can further explore the interactions and fine-tuned regulation of EVs in physiological and pathological processes and improve the accuracy of clinical liquid biopsies. Thus, to fully harness the potential of EVs, it is crucial to advance EV subpopulation separation and detection methods.

## Separation methods for EVs

### Traditional methods for EV separation

Traditional methods for EV separation primarily rely on fundamental biophysical properties such as density and particle size. Differential ultracentrifugation is the most widely used method and is considered the “gold standard” for EV separation 45. This technique involves sequentially increasing centrifugation speeds to separate EVs from large volumes of biological samples. Another commonly used traditional method is density gradient centrifugation (DGC), which employs a density gradient, typically created with sucrose or iodixanol for separation 46. Both differential ultracentrifugation and DGC depend on the density of EVs for separation. Size exclusion chromatography (SEC) and ultrafiltration (UF) separate EVs based on differences in particle size relative to other impurities 47,48. Precipitation methods involve adding polymer such as polyethylene glycol (PEG) to alter the solubility of EVs, allowing their separation through low-speed centrifugation 49. Research indicates that, combining traditional methods like ultracentrifugation, UF, and SEC can significantly enhance the purity of the separation 50. Modifying separation equipment and reducing blockage rates can also improve the thoroughness of these processes. However, these methods are often time-consuming, may leave behind substantial impurities, and are challenging to apply to small sample volumes. Additionally, they might damage the biological activity of EVs. While these methods can separate different EV subpopulations based on density and particle size, they struggle with the heterogeneity of EV cargo. Traditional methods also include biochemical property-based methods, such as immunoaffinity techniques, which utilize antibodies targeting specific tetraspanins on EVs 51. However, they too fail to separate EV subpopulations with distinct cargo. Likewise, although some commercial kits have been available for a long time, most fail to address these issues effectively as well (**Table 1**).

### Burgeoning methods for EV separation

In recent years, several burgeoning methods have significantly advanced EV research. For example, some microfluidic technologies exploit the unique physical properties of EVs, such as electrical, acoustic, or fluidic characteristics, to improve separation efficiency and purity, enabling rapid EV separation and characterization 52-54. Moreover, covalent chemistry methods demonstrate rapid capture and release of EVs, potentially preserving their biological activity and offering new avenues for downstream applications 55. However, single burgeoning separation methods cannot achieve high purity of EV subpopulations typically. Despite this, burgeoning methods have specific advantages over traditional techniques, like improved separation efficiency, purity and elevated biological activity (**Table 1**).

## Separation methods for EV subpopulations

To selectively separate specific EV subpopulations, most methods rely on immunoseparation techniques. These approaches typically use antibodies or nucleic acid aptamers to capture specific proteins on the EV membrane. Researchers often exploit the high affinity between biotin and streptavidin to link antibodies or aptamers to a solid substrate or magnetic beads, thereby enabling the separation of specific EV subpopulations from samples. Additionally, these methods are often combined with other separation strategies to enhance separation efficiency and specificity (**Table 2**).

### Microfluidic chip-based subpopulation separation methods: a promising pathway for clinical diagnostics

Microfluidic chip technology offers an innovative approach to separate EVs by manipulating and processing small fluid volumes within a chip. Early microfluidic chips often targeted EV markers, such as CD9, CD63, and CD81, to separate non-specific EVs, with anti-CD63 antibodies frequently employed to capture EVs from various cell sources 51,56. However, recent years have witnessed the emergence of its hidden potential for EV subpopulation separation.

**Figure 2:**
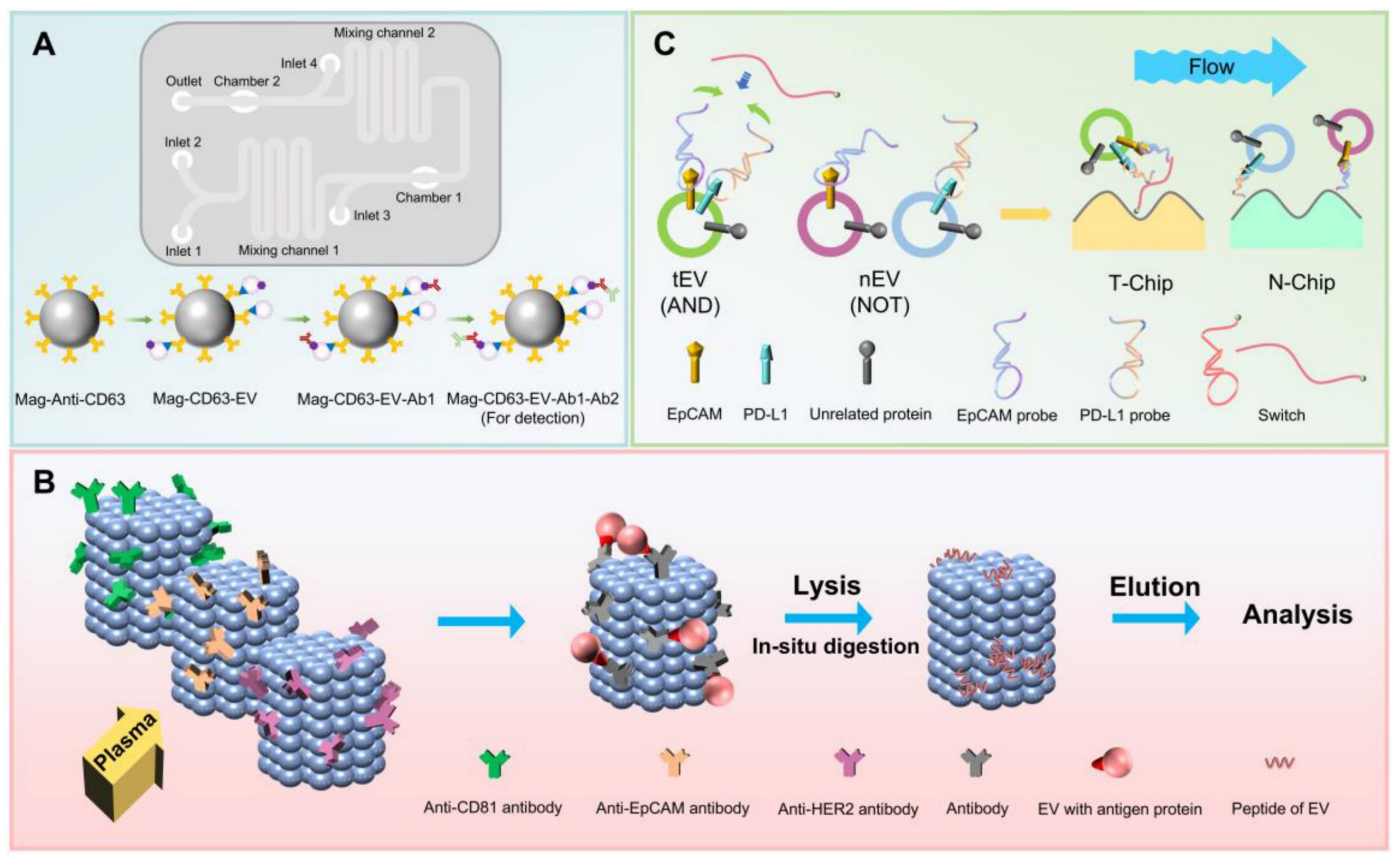
Microfluidic chip-based subpopulation separation methods. (A) The on-chip capture microfluidic device. (B) Separation of single-positive and double-positive EV subpopulations using AND and NOT logic algorithm. (C) The Sub-ExoProfile chip-based EV subpopulations separation and proteins profiling.

Researchers have developed a microfluidic device for on-chip capture of specific EV subpopulations from cell culture media or patient plasma 57 (**Figure 2A**). The chip features four inlets, one outlet, and two chambers for collecting immunomagnetic particles. Plasma is introduced into the microfluidic chip and pre-mixed with magnetic nanoparticles linked to anti-CD63 antibody (Mag-Anti-CD63) to filter EVs, enabling the formation of Mag-CD63-EV complexes. As the sample flows through the chip, these complexes interact with selective primary antibodies and fluorescently-labeled secondary antibodies, forming Mag-CD63-EV-antibody (Ab) 1-Ab2 complexes detected by fluorescence. By selecting specific primary antibodies, the chip can capture EpCAM^+^ and HER2^+^ EVs, and successfully detect fluorescently-labeled EV complexes. The use of polymer coatings for magnetic nanoparticles, as opposed to gold-coated nanoparticles, prevents fluorescence signal quenching, enhancing detection sensitivity. Overall, this method is simple, time-efficient, cost-effective, minimally damaging to EVs, and supports breast cancer diagnosis and molecular subtyping. However, it should be noted that, this approach represents a conventional microfluidic method with limited novelty.

For breast cancer clinical detection, researchers have developed a chip named Sub-ExoProfile 58 (**Figure 2B**). This chip incorporates a three-dimensional (3D) nanopillar structure assembled from amphiphilic mesoporous silica nanoparticles (SiNPs) carrying anti-CD81, anti-EpCAM, and anti-HER2 antibodies to separate total EVs, tumor-derived EVs, and breast cancer EVs in the same flow channel. By combining biological markers, this design enables high-throughput separation of three specific EV subpopulations. The three nanopillars are individually controlled by three valves and separated from each other. The 3D nanoporous structure enhances interface binding efficiency, achieving nearly 100% recovery of EV subpopulations, even for those with low surface marker expression. SiNPs can be used not only for breast cancer clinical detection but also as nanoreactors for protein digestion enrichment and analysis of subpopulation protein expression differences, facilitating the study of EV subpopulations in cancer development and metabolism, though this method cannot obtain single undamaged EVs for downstream analysis.

As a promising clinical diagnostic approach, EV subpopulations processed by microfluidic chips require intelligent and efficient detection methods. The rapid development of DNA computing, particularly through aptamer recognition, provides strong support for EV subpopulation separation and detection. Due to the complexity of logical design and high EV heterogeneity, most methods are limited to quantifying specific EV subpopulations.

To address this, researchers have developed a modular platform that integrates AND and NOT logic algorithm, utilizing DNA computing-mediated microfluidic serial methods to successfully separate EpCAM and PD-L1 double-positive EV subpopulations 59 (**Figure 2C**). The platform employs aptamer probes with extended regions, where only EVs co-expressing EpCAM and PD-L1 can induce the proximity of both probes. Increased concentrations of probe extension sequences activate switch probes with a hairpin structure. The latch motif sequence of the switch probe isolates the complementary DNA from the extension region. By adjusting the lengths of the switch probe latch domains and probe extensions, the thermodynamic balance is altered so that the probe is recognized and activated only after binding to both affinity probes, achieving optimal hybridization balance. Through combined recognition-mediated switch activation, biotin on the switch probe captures tumor-derived PD-L1^+^ EVs on a streptavidin-functionalized microfluidic chip, referred to as the T-chip. EVs expressing only PD-L1 bind to PD-L1 probes, and the extension sequence on the probe hybridizes with complementary DNA modified on a separated microfluidic chip, termed the N-chip, thereby separating this EV subpopulation. Both chips can release EVs through nuclease degradation of aptamers without significantly altering protein integrity, allowing for subsequent protein analysis, such as proteomics, and clinical applications, such as immune evaluation.

### Click chemistry/covalent chemistry-based subpopulation separation methods: a pathway for separating bioactive subpopulations

Click chemistry represents a class of widely recognized and convenient chemical transformations that selectively promote molecular construction 60. The classic click ligand reaction, copper-catalyzed azide-alkyne cycloaddition, was developed by Sharpless and Meldal 61. However, the triazole nature and the inherent toxicity of copper limit its application in biological systems. Bertozzi's development of two-component click ligand reactions effectively addressed these issues, marking the advent of bioorthogonal click chemistry 62. In this field, new methods have continued to emerge, such as the inverse electron demand Diels-Alder (IEDDA) reaction and Staudinger ligation reactions, further advancing the field of click chemistry 63.

**Figure 3:**
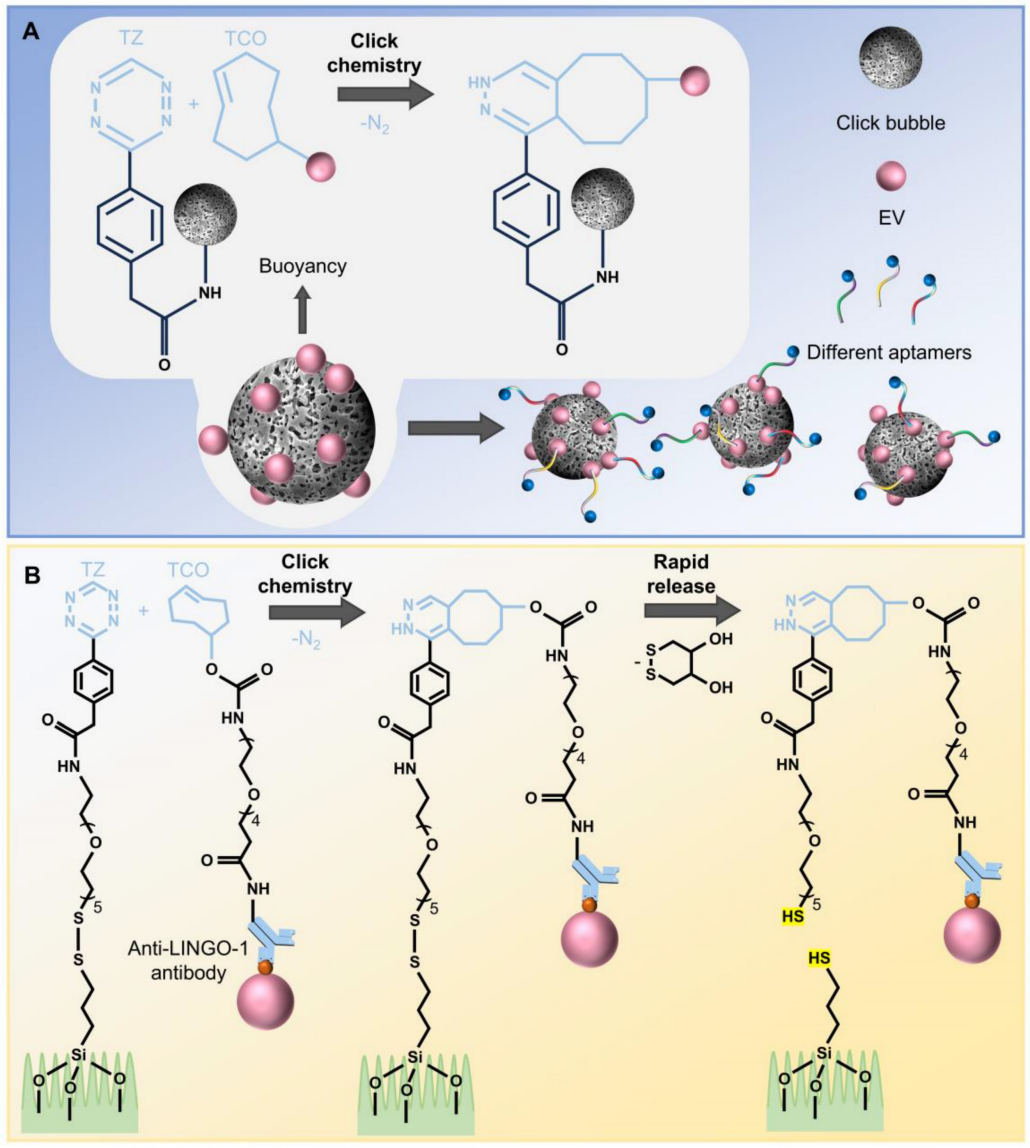
Click chemistry/covalent chemistry-based subpopulation separation methods. (A) Click-bubble-based EV subpopulation separation and fluorescent aptamer binding, facilitated by flipping and buoyancy-induced bubble self-aggregation. (B) LINGO-1^+^ EV separation mediated by click chemistry between Tz and TCO.

In recent years, the application of click chemistry to EV subpopulation separation has gained incremental attention. Researchers have utilized the click chemistry reaction between tetrazine (Tz) and trans-cyclooctene (TCO) to separate EVs on click bubbles 64 (**Figure 3A**). In this approach, hollow bubble@amyloid-like phase transition of bovine serum albumin@Tz, the click bubble, is synthesized. Through the click chemistry reaction between Tz and TCO, multiple TCO-labeled EVs are immobilized onto the click bubbles. By mixing these with different fluorescently labeled aptamers and using fluorescence detection, differences in surface protein markers of EVs from breast cancer, liver cancer, and lung cancer patients compared to healthy donors can be explored. This method enables classification based on the distinct protein profiles of different cancers. Meanwhile, the click bubbles are engineered to enter a “supervalent state”, facilitating the rapid enrichment of EVs through click reactions. The buoyancy of the click bubbles causes them to aggregate at the top of the liquid droplet, reducing background interference during fluorescence analysis and enabling efficient separation. However, this strategy primarily separates EVs from the sample and explores membrane protein heterogeneity, failing to achieve true subpopulation separation or maintain the good biological activity of EVs for downstream studies. Moreover, there is a potential risk of fusion between multiple EVs during the process.

Researchers have also developed a novel Ewing sarcoma (ES) EV Click Chip for the purification and functional study of sarcoma-derived EVs by combining covalent chemistry 65 (**Figure 3B**). In this system, Tz and TCO are introduced as highly reactive click chemistry groups. Tz is surface-modified and bound to the silicon nanowire substrate (SiNWS) of the chip, while TCO is conjugated with the anti- leucine-rich repeat and immunoglobulin-like domain-containing protein 1 (LINGO-1) antibody through the N-hydroxysuccinimide ester reaction, forming a TCO-anti-LINGO-1 conjugate. Due to the rapid IEDDA cycloaddition between Tz and TCO, the TCO-anti-LINGO-1 conjugate linked to ES-EVs is rapidly, selectively, and irreversibly captured on the Tz-modified SiNWS. The disulfide bond cleaving agent, 1, 4-dithiothreitol (DTT), can break the disulfide bonds embedded in Tz-SiNWS, allowing rapid release of ES-EVs. The nanostructures within the chip significantly increase the surface area for contact with ES-EVs, enabling efficient separation of ES-EVs with low antibody consumption. The usage of DTT as a relatively mild reagent, facilitates rapid release while preserving the integrity and biological activity of EVs, allowing for downstream analysis of their physiological and pathological roles in intercellular communication.

### Two aptamer-based subpopulation separation methods: innovative separation approaches

**Figure 4:**
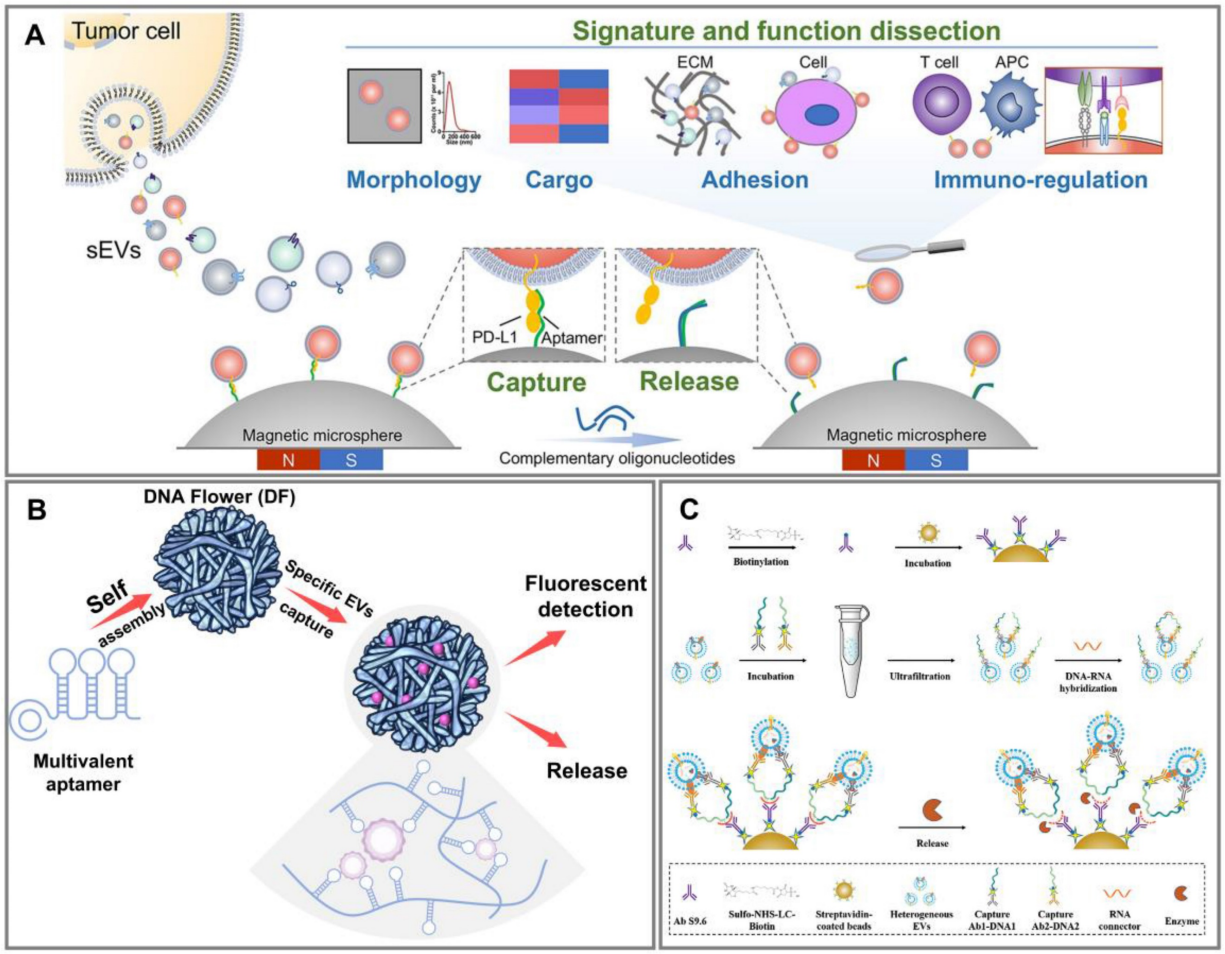
Aptamer-based subpopulation separation methods. (A) Traceless separation of PD-L1^+^ EV for accurate dissection of its subpopulation signature and function. Adapted with permission from 16, copyright 2023 American Chemical Society. (B) EpCAM^+^ EV separation and subsequent applications based on DFs. (C) Robust separation and release of EV with double-positive membrane protein. Adapted with permission from 73, copyright 2024 John Wiley and Sons Ltd.

Aptamers are small, single-stranded DNA or RNA molecules that, by folding into specific three-dimensional structures, exhibit strong affinity and selectivity for their specific ligands, enabling them to bind to particular targets 66,67 (**Figure 4A**). Due to their functional similarity to antibodies, aptamers are also referred to as “chemical antibodies” 68. However, aptamers offer several advantages over antibodies. They are smaller in size and have a wider range of potential targets 69. The specific 3D structure of aptamers facilitates the formation of aptamer-target complexes 70, conferring comparable binding affinity and specificity, with even superior advantages over antibodies 69. Through sequence design, nanostructure design, and multifunctional integration, the binding strength of aptamers to proteins can be fine-tuned, making them highly promising for targeted subpopulation separation 71. More importantly, aptamers can be degraded by nucleases, enabling the reversible release of separated EV subpopulations, which is a key advantage in the field of EV subpopulation separation compared to antibodies, facilitating non-destructive separation. The small size, broad target range, high affinity, and high versatility of aptamers make them promising, creative, and innovative tools for EV subpopulation separation.

#### Multivalent DNA nanoflowers

Researchers have developed multivalent DNA nanoflowers based on rolling circle amplification (RCA) technology for the efficient separation and detection of EpCAM^+^ EVs, allowing their release for downstream studies 72 (**Figure 4B**). Initially, single-stranded DNA molecules containing multiple EpCAM fragments are generated through RCA. Using an anisotropic DNA liquid crystal process, these DNA molecules self-assemble into a dense floral DNA/magnesium pyrophosphate hybrid complex, ultimately forming micron-sized DNA flowers (DFs) through a one-pot reaction. These DFs are capable of directly capturing EpCAM^+^ EVs from samples. The formed EpCAM^+^ EVs-DFs complexes can be collected via low-speed centrifugation. The EpCAM^+^ EVs can then be used for liquid biopsy due to dye labeling. Since DFs are assembled from aptamer molecules, they can be degraded by deoxyribonuclease I, releasing the EVs while maintaining good integrity and high biological activity. Common monovalent aptamers exhibit weak affinity and may entangle, altering their biological activity and reducing effective interaction and capture efficiency. In contrast, DFs utilize a multivalent aptamer-receptor system, with multiple binding sites within a limited area, enhancing the local concentration of aptamers and increases the effective surface area, thereby significantly increasing affinity and capture efficiency. Moreover, this method requires no chemical modification or solid material surface fixation, and EVs can be separated from samples by simple centrifugation. However, this approach can only effectively distinguish cancer patients from healthy individuals and cannot classify different types of cancer.

#### Robust separation of double-positive EVs

Currently, most existing methods for EV subpopulation separation struggle to target multiple positive markers simultaneously, or they target more than one marker in a crude manner, leading to relatively low accuracy. However, accurately identifying these markers is crucial for understanding disease progression and improving therapeutic outcomes. Moreover, separating EV subpopulations that express multiple positive markers is particularly beneficial for precise cancer diagnosis, prognosis, and exploring the biological significance of specific EVs.

To address this, researchers have developed a double-positive EV subpopulation separation and enrichment method based on the proximity ligation assay 73 (**Figure 4C**). This method uses a pair of capture antibody-DNA (Capture Ab1-DNA1/Capture Ab2-DNA2) conjugates, where the capture antibodies and DNA are connected through biotin and streptavidin. During incubation with the sample, these conjugates recognize and bind to different proteins on the same EV, causing the nucleic acid probes to produce a proximity effect and approach each other, converting protein signals into nucleic acid signals. Subsequently, the addition of an RNA connector closely links the DNA ends of the two conjugates, forming a DNA-RNA hybrid. The RNA connector is designed to a specific length, so the DNA-RNA hybrid can form only when the two conjugates bind to the same EV. The antibody S9.6 specifically recognizes DNA-RNA hybrids and, through biotin and streptavidin, binds to magnetic beads, linking with the DNA-RNA hybrid in the system. Ultimately, double-positive EVs are separated using an external magnetic field. Deoxyribonuclease I or ribonuclease A can degrade the DNA-RNA hybrid, releasing and enriching EVs of classical size ranges, although the concentration of EVs obtained is significantly lower than the initial state. This method provides a simple and effective route for separating double-positive membrane protein EV subpopulations with good specificity, minimizing interference from soluble proteins.

### Subpopulation separation method not based on antibodies or aptamers: unique but less versatile approach

**Figure 5:**
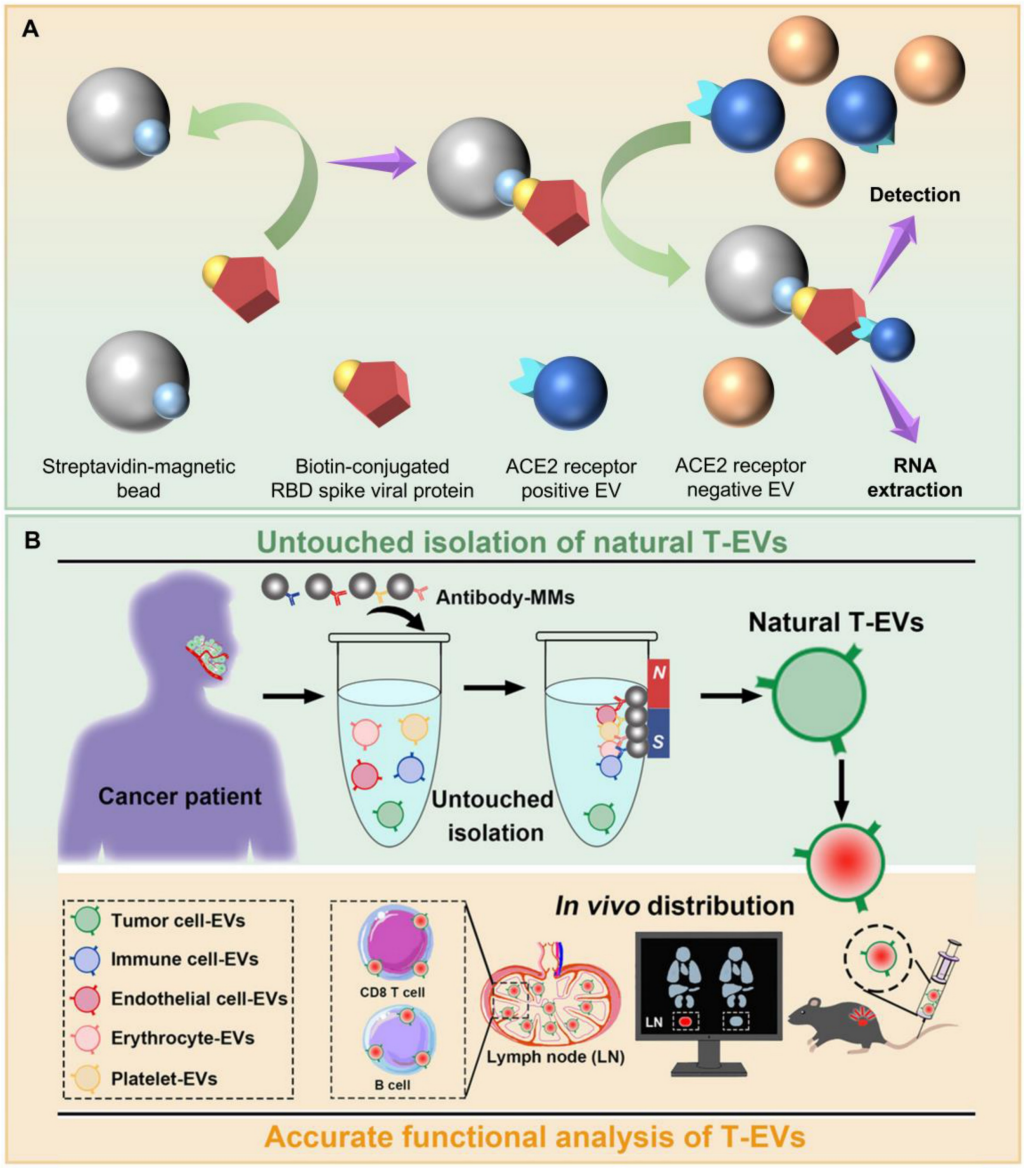
Subpopulation separation methods not based on antibodies or aptamers and based on negative strategy. (A) ACE2^+^ EV separation based on receptor-ligand interaction between ACE2 receptor and RBD spike viral protein. (B) Natural tumor-cell derived EV separation by removing non-tumor-cell derived EVs. Adapted with permission from 75, copyright 2022 Taylor and Francis Ltd.

A novel method has been developed to extract angiotensin converting enzyme 2 (ACE2) positive EVs from the body fluids of patients with severe acute respiratory syndrome coronavirus 2 and to study their microRNA (miRNA) molecules 74 (**Figure 5A**). Initially, total EVs are captured using anti-CD63 antibody, and ACE2 expression in samples is confirmed with anti-ACE2 antibodies. Subsequently, recombinant biotin-conjugated receptor binding domain (RBD) spike viral protein is conjugated with streptavidin-coated magnetic beads and mixed with the samples. Through receptor-ligand interactions, the RBD spike viral protein binds to ACE2, allowing ACE2^+^ EVs to associate with both the RBD spike viral protein and, consequently, with the magnetic beads. This complex can then be separated using an external magnet. The study further separates ACE2^+^ EV subpopulations from different patients to amplify and analyze their miRNA. Results demonstrate upregulation, downregulation, and dysregulation of miRNA between ACE2^+^ EV subpopulations and ACE2^-^ EV subpopulations, highlighting the significant role of EVs in pathological conditions.

### Subpopulation separation methods based on negative separation strategy: a step toward theoretically non-destructive approach

Presently, most methods for separating EV subpopulations inevitably require direct contact between the separation materials and target EVs. This strategy allows for the selective separation of EVs carrying specific surface markers, with relatively minimal impact on liquid biopsy samples. However, this approach has inherent limitations in downstream mechanistic studies. For example, it can only target positive EVs with surface markers and is not suitable for obtaining negative EVs. Since antibody-based separation strategies often struggle to separate positive EVs without altering their characteristics, the natural physical properties of EVs, such as quality and morphology, can be modified, leading to alteration of their biological activity. Additionally, surface markers on EVs may be masked by antibodies, which can result in further influence on their biological functions.

Although aptamer-based separation strategies can sometimes preserve certain physical characteristics and biological activities of EVs by releasing them through nuclease degradation of aptamers, the binding and separation process can more or less impact the original state of the EVs. Furthermore, EVs derived from *in vitro* cultured cell lines lack interactions with other cells and molecules, making it challenging to replicate the dynamic and natural state of *in vivo* environments.

To address these challenges, researchers have developed a non-contact separation strategy that selectively separates tumor tissue-derived EVs (T-EVs) from patients with oral squamous cell carcinoma 75 (**Figure 5B**). T-EVs exhibit high heterogeneity, with a significant proportion originating from immune cells compared to tumor cells, along with contributions from endothelial cells, red blood cells, and platelets. By constructing and using capture antibodies (including anti-CD3 antibody, anti-CD11b antibody, anti-CD45 antibody for immune cells; anti-CD144 antibody for endothelial cells; anti-CD235a antibody for red blood cells; anti-CD41 antibody for platelets) coupled with magnetic microparticles, EVs from non-tumor cell sources in the samples are removed, resulting in the separation of tumor cell-derived, specific EVs with preserved natural characteristics.

The most prominent advantage of this strategy is its ability to preserve the natural properties of T-EVs to the greatest extent, with minimal impact on the physical properties and biological functions of the target EV subpopulations, such as cargo composition, *in vivo* dynamics, and distribution performance. This approach also allows for the separation of EV subpopulations from other sources by simply adjusting the types of antibodies used, providing a new avenue for non-damaging separation of EV subpopulations and facilitating downstream research on the biological functions of specific EVs. One minor deficiency of this strategy is the necessity for a clear understanding of non-target subpopulations and the extensive use of relevant antibodies; otherwise, residual negative subpopulations can easily persist, resulting in lower purity.

## Detection methods for EV subpopulations

For downstream research, a major issue in the EV field is how to effectively separate distinct EV subpopulations prior to detection, as traditional detection methods are capable of providing accurate quantitative analysis. However, in the context of clinical liquid biopsy, the situation may differ. In some cases, EV subpopulations do not need to be quantitatively analyzed but rather qualitatively or semi-quantitatively, and these subpopulations can be detected without prior separation. In such cases, rapid, accurate, and high-throughput detection methods are particularly valuable.

The key to detecting EV subpopulations lies in transforming the biological signals of specific molecules carried by EVs into other detectable signals, such as optical signals with color/fluorescence changes or electrochemical signals reflecting variations in ion concentration, which allows for detection by the naked eye or automated devices. Traditional detection methods, such as enzyme-linked immunosorbent assay (ELISA) and fluorescence dye detection, have been applied in some EV subpopulation separation methods. However, the former lacks sensitivity compared to advanced methods while the latter suffers from lower specificity, making it challenging for precise detection of EV subpopulations 76. In contrast, advanced detection methods offer significant advantages. For example, optical signal-based detection is typically more convenient, rapid, and requires smaller sample volumes, while electrochemical signal-based detection excels in detection range, precision, and sensitivity. The ongoing development of EV subpopulation detection methods is paving the way for new insights and opportunities for clinical liquid biopsies (**Table 3**).

### Nanoparticle flow cytometry (NanoFCM): widely applied strategy

Flow cytometry forms the foundation for the development of NanoFCM. Traditional flow cytometry involves the measurement of cells or particles in flow, where cells must be in suspension 77. The cells are focused into a single line, clearly separated from one another 78, allowing for individual analysis even at high flow velocities. This technique detects fluorescence and light scattering signals from thousands of individual particles per second within a fluid stream 79. Over the years, various advanced forms of flow cytometry have emerged, including spectral flow cytometry and mass cytometry, which address challenges such as spectral overlap and even use metal isotopes instead of traditional fluorochromes for labeling cells 80,81. However, these traditional flow cytometry methods are not well-suited for EVs, which are much smaller than cells. EVs often generate signals that overlap with background noise, making it difficult to detect them accurately using traditional flow cytometry techniques. This is where NanoFCM becomes particularly valuable. The basic principle of NanoFCM is similar to that of traditional flow cytometry, but it distinguishes itself by analyzing individual EVs as they pass through the system. This enables NanoFCM to detect differences in the cargo carried by isolated EVs, facilitating the identification of distinct EV subpopulations.

NanoFCM has already been widely applied to detect EV subpopulations in various contexts. For example, it has been used to identify prostate cancer patient plasma-derived six-transmembrane epithelial antigen of the prostate 1-positive EV subpopulations 82, pluripotent stem cell-derived sEV subpopulations 83, EGFR-positive EV subpopulations from glioblastoma cells 84, and dual-positive EGFR and CD9 EV subpopulations from human colorectal cancer cells 85. It has also been used to analyze EV subpopulations with diverse DNA cargo 86. In recent years, significant improvements have been made to NanoFCM, including simplifications and modifications to detection strategies 87-89 and optimization of device parameters 90. Although NanoFCM has been widely applied and enhanced for high-throughput quantitative and qualitative analysis, challenges remain, particularly when detecting smaller EV subpopulations. One of the main issues is overcoming the interference from noise signals 91. Further advancements are still needed to refine its capabilities and expand its potential in EV research.

### Color/Fluorescence reaction-based optical subpopulation detection methods: convenient and rapid detection strategy

#### Single molecule array (Simoa)

Single molecule arrays represent an advanced technology for single-molecule protein detection with sensitivity far exceeding that of ELISA 92,93. This approach involves immobilizing a large number of antibodies onto magnetic beads, where each bead is designed to capture either none or at most one target molecule to form an immune complex. Streptavidin-β-Galactosidase (SβG) is conjugated with another biotin-labeled detection antibody for the target molecule, forming a capture antibody-target molecule-biotinylated detection antibody-SβG complex on the beads. The galactosidase in SβG hydrolyzes the substrate resorufin β-d-galactopyranoside (RGP), generating a fluorescent product for single-molecule detection, which enables rapid detection of EV subpopulations.

**Figure 6:**
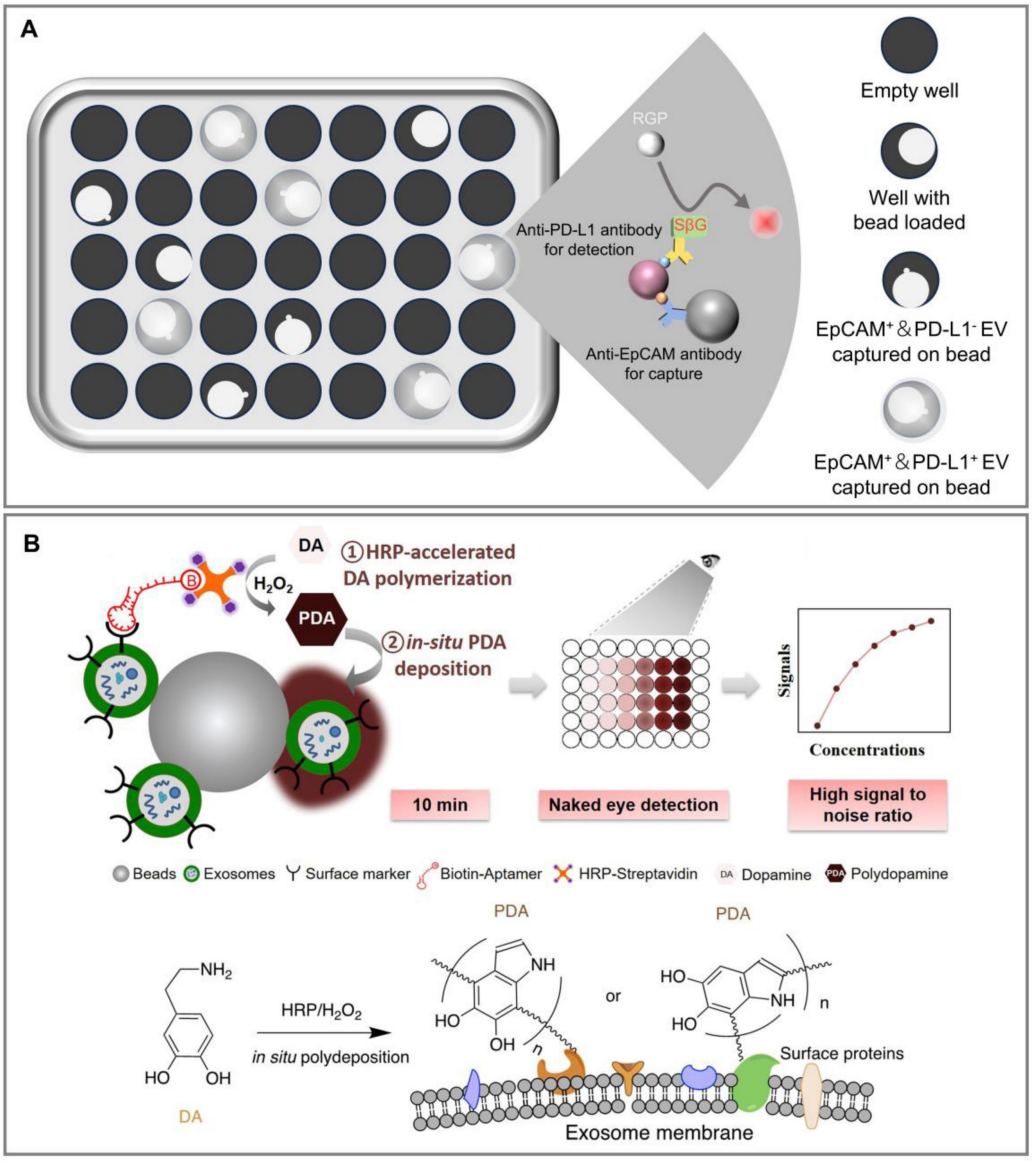
Color/Fluorescence reaction-based optical subpopulation detection methods. (A) Simoa immunoassay for detecting EpCAM and PD-L1 double-positive EV subpopulation by the increasing fluorescent signal from the catalytic reaction of SBG with RGP, confined within the micro-well. (B) The ExoAptaSensor with HRP accelerated dopamine polymerization and deposition for EV subpopulation detection. Adapted with permission from 95, copyright 2020 Elsevier Ltd.

In the context of high-sensitivity detection of plasma EVs from cancer patients and tumor-derived EVs, the Simoa method has been applied 94 (**Figure 6A**). Researchers utilize anti-EpCAM and anti-PD-L1 antibodies as capture and detection antibodies respectively, and incubate the sample with magnetic beads coated with anti-EpCAM capture antibodies, forming magnetic bead-anti-EpCAM antibody-EV complexes. These complexes are then sequentially incubated with biotinylated anti-PD-L1 detection antibodies and SβG. The final complex is mixed with RGP and load into over 20,000 single molecule arrays, encased in an oil layer for fluorescent imaging. Analysis software calculates the ratio of wells containing both beads and fluorescent signals to the total number of wells containing beads, determining the average per bead, which corresponds to the Simoa signal. This method allows for the complete automation of EpCAM^+^ EVs separation and enables precisely detection of EpCAM and PD-L1 dual-positive EVs with high sensitivity and throughput, thereby facilitating the detection of clinical biomarkers. Although the method's applicability for detecting tumor EVs requires further validation due to the limited patient sample, it shows potential for non-invasive, dynamic detection of EpCAM and PD-L1 dual-positive EVs in patients undergoing immune checkpoint inhibitor therapy. However, a limitation is that this strategy can only be used for fluorescence detection of dual-positive subpopulations, which may hinder its application in downstream research.

#### Horseradish peroxidase (HRP)-mediated color/fluorescence reaction

Aptamer-based colorimetric sensors have been used for the specific detection of target EVs 95 (**Figure 6B**). Through aldehyde-imine condensation, researchers anchor Aldehyde latex microbeads to EVs, then sequentially add biotinylated specific antibodies and streptavidin-conjugated HRP, forming a Bead-EV-Aptamer-HRP complex. In the presence of the oxidant hydrogen peroxide, HRP can catalyze the color reaction of the colorless dopamine (DA) solution, producing a colored product, polydopamine (PDA), after polymerization. PDA reacts well with amino, sulfhydryl, and phenolic groups on the EV surface, allowing itself to deposit on EVs. Absorbance measurements provide a signal that is linearly related to the logarithm of the concentration of EVs expressing specific proteins (e.g., HER2). The color change can be semi-quantitatively or qualitatively analyzed by the naked eye, or quantitatively by measuring absorbance. This method overcomes the limitations of detecting on solid substrates by using a solution format, significantly improving sensitivity. The method can be completed in minutes, with advantages of low sample demand and cost.

It is noteworthy that HRP, as a peroxidase enzyme, can catalyze not only the conversion of DA to PDA, but also other colorimetric reactions using substrates such as 2,2′-Azino-bis (3-ethylbenzothiazoline-6-sulfonic acid) and 3,3′,5,5′-Tetramethylbenzidine 96. Moreover, by substituting the substrate with compounds like Amplex Red or thiamine, HRP can also mediate fluorescence reactions 96. While these substrates are less frequently applied in EV subpopulation detection, they offer valuable versatility for a range of assay formats.

### Electrochemical sensor subpopulation detection method based on ion concentration changes: precise and sensitive detection strategy

**Figure 7:**
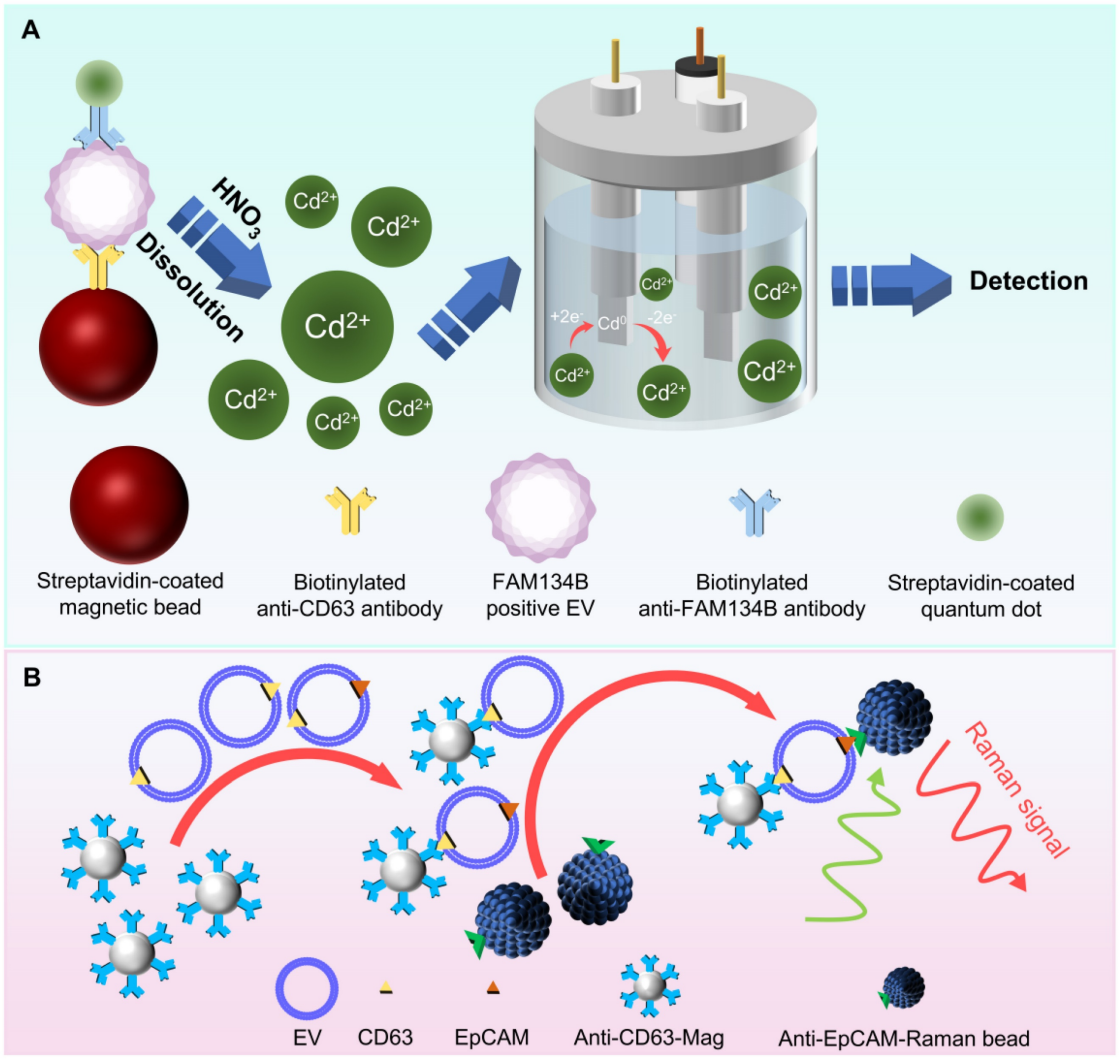
Electrochemical sensor-based and Raman bead-based subpopulation detection methods. (A) The assay for electrochemical detecting FAM134B^+^ EV using cadmium-containing quantum dots. (B) Detection of EpCAM^+^ EV using Raman beads.

Researchers have developed an electrochemical method using quantum dots as signal amplifiers to separate and detect EV subpopulations through stripping voltammetry 97 (**Figure 7A**). In this approach, the sample is first mixed with anti-CD9/CD63-functionalized magnetic beads, allowing antibodies to bind with EVs. Following this, CdSe quantum dot (QD)-functionalized biotinylated anti-HER-2 or anti-family with sequence similarity 134 member B (FAM134B) antibodies, targeting EVs related to breast cancer and colon cancer, respectively, are added. This leads to the formation of a magnetic bead-CD9/CD63-EV-HER-2/FAM134B-CdSeQD immune complex, which is subsequently dissolved in nitric acid solution to release Cd^2+^ ions for analysis. Square-wave anodic stripping voltammetry is performed using a three-electrode system (glassy carbon working electrode, Pt counter electrode, and Ag/AgCl reference electrode).

By constructing an EV concentration gradient, it is found that the peak current associated with the stripping of Cd2^+^ is proportional to the number of specific EVs, showing good reproducibility. Due to the signal amplification effect of quantum dots, this method can enhance the sensitivity of the immunoassay by hundreds of times, requiring only 10 μL of sample compared to the minimum 100 μL required for traditional ELISA, effectively addressing the issue of low EV concentrations in very small clinical samples. This study employs magnetic separation methods to mitigate the limitations of traditional antibody fixation on sensor surfaces, offering a simpler and relatively rapid binding approach.

### Raman beads: a promising detection strategy

Raman scattering is an inelastic scattering phenomenon where the wavelength of light changes 98. Raman spectroscopy plays a significant role not only in characterizing two-dimensional materials and phonon modes in crystals, but also has had a profound impact in biological and medical fields 99-102. It is a label-free, chemically selective hyperspectral imaging technique that can non-invasively detect molecular composition and concentration gradients 103,104. However, the inherently weak Raman signal limits its practical applications. By placing the analyte on a roughened metal surface, the measured Raman signal can be amplified by several orders of magnitude, a phenomenon known as surface-enhanced Raman scattering (SERS), which is widely used to enhance Raman signals 105-107. However, despite its effectiveness, SERS is still constrained by the requirement of a metal substrate.

To overcome these limitations, Raman beads have been developed, which are made from novel Raman-active polymeric nanomaterials that exhibit strong and unique Raman vibrations within the biological Raman silent region (1800-2800 cm⁻¹) 108,109. These beads overcome the limitations of specific substrates, serving as biomarkers for detecting specific substances, including EV subpopulations.

Researchers have developed a microfluidic Raman biochip for the effective diagnosis of prostate cancer 110 (**Figure 7B**). First, magnetic beads conjugated with anti-CD63 antibodies and EpCAM-functionalized Raman beads are prepared. EV samples and anti-CD63 magnetic beads are pumped into the Raman chip, where they mix and react in the staggered triangular micropillar mixing chamber to form a Mag-CD63-EV complex. This complex is flushed from the mixing chamber with phosphate buffered saline and retained in the Raman detection zone under an applied magnetic field. Subsequently, EpCAM-functionalized Raman beads are introduced, binding with the EVs to form a sandwich complex, which is finally detected by Raman spectroscopy.

Experimental results demonstrate a good linear relationship between the measured Raman signal intensity and the logarithmic value of the EV concentration. The chip offers several advantages. First, the triangular micropillar array used as a micromixer facilitates thorough solution mixing, thereby improving EV capture efficiency. Additionally, since lipids, proteins, and nucleic acids exhibit Raman activity in the ranges of 400-1800 cm⁻¹ and 2800-3100 cm⁻¹, and the Raman peak at 2230 cm⁻¹ lies within the Raman silent region, choosing this region as the quantitative signal eliminates interference from complex sample components. By comparing the serum of clinical prostate cancer patients with that of healthy individuals, it is found that the former has significantly higher numbers of EpCAM^+^ EVs. This device effectively distinguishes between the two types of serum samples, providing a potential solution for the rapid screening and preliminary diagnosis of prostate cancer.

## Challenges and future prospects

**Figure 8:**
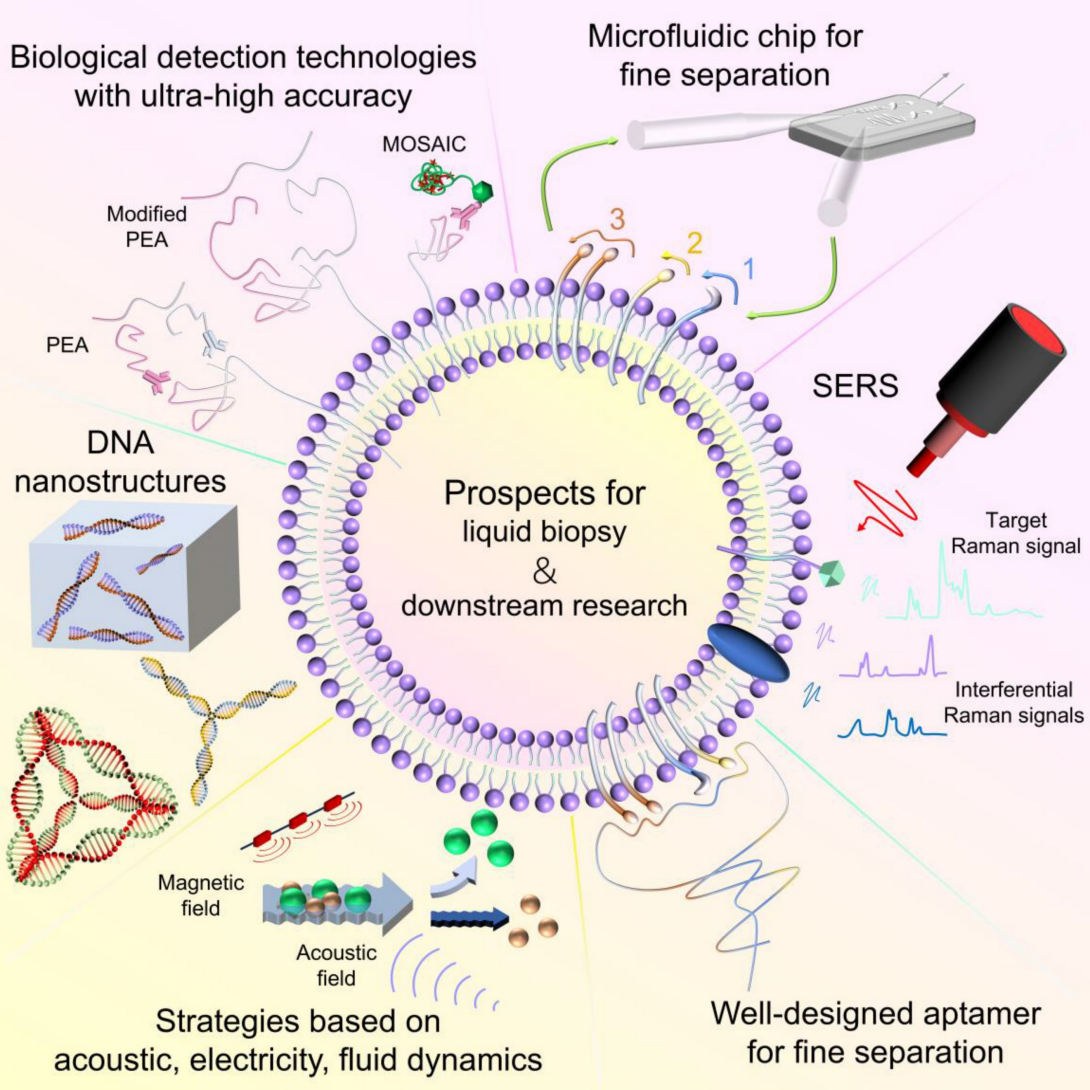
EV subpopulation separation and detection prospects for liquid biopsy and downstream research, including SERS, new microfluidic chips for fine separation, strategies based on biological detection technologies with ultra-high accuracy, for liquid biopsy; and DNA nanostructures, well-designed aptamer for fine separation, strategies based on acoustic, electricity, fluid dynamics, for downstream research.

Although the separation and detection of EV subpopulations have gradually gained attention and made progress, several concerning issues remain unresolved. Therefore, we are proposing some potential prospects to help researchers avoid pitfalls and missteps when designing novel strategies for clinical liquid biopsy or downstream clinical research, as outlined below (**Figure 8**).

### Clinical liquid biopsy

In clinical liquid biopsy, most captured EV subpopulations do not require further release. The core focus of subpopulation separation and detection lies in its accuracy, reproducibility, sensitivity, and rapidity. Although some studies have attempted to separate EV subpopulations through ligand-receptor binding, the differing affinities and specificities of various ligand-receptor pairs have led to the prevalent use of highly specific antibodies and aptamers for subpopulation separation. We believe these tools provide adequate accuracy for separating EV subpopulation markers. However, they do have certain limitations.

#### Multiple reaction steps resulting in limited separation efficiency and speed

The main disadvantage of using antibodies or aptamers is that they cannot be directly used for detection. As a result, additional reaction steps are required to detect the antibody/aptamer-EV complex, thereby identifying the presence or quantifying the amount of EV subpopulations. Current detection methods include: (1) Lysis and on-site digestion followed by peptide analysis; (2) An ELISA-like approach, using a detection antibody targeting the capture antibody and conducting a chromogenic reaction with the substrate carried by the detection antibody; (3) Labeling antibodies or aptamers with fluorescent molecules, enzyme-containing molecules, metal ion-releasing molecules, or Raman beads for detection through fluorescence imaging, catalytic color reactions, electrochemical methods, or Raman spectroscopy. Most of these methods involve multiple reaction steps, each of which is difficult to achieve complete reaction. A failure in any step results in undetectable outcomes, causing quantitative detection of EV subpopulations to fall below actual values.

To address this issue, we have proposed a future research direction: utilizing a label-free strategy based on SERS to detect EV subpopulation markers, thereby achieving direct detection that closely matches the actual concentration of EV subpopulations while minimizing interference from other bioactive substances. This integrated separation and detection strategy also enhances the rapidity of liquid biopsy. Furthermore, by incorporating materials and structures such as ultrasmall Fe_3_O_4_ nanoparticles 111, octahedral Cu_2_O@Ag particles 112, homogeneous flower-like Cu_2_O@Ag composites 113, amorphous nitrogen-doped carbon nanocages 114, Fe_2_O_3_@CeO_2_ heterojunction substrates 115, octahedral Ag_2_O nanoparticles 116, or with the assistance of machine learning 117, significant improvements in SERS activity, sensitivity, and stability can be achieved. These advancements are advantageous for accurate clinical disease diagnosis. However, the application of SERS technology in EV subpopulation-based clinical liquid biopsy remains limited, representing a promising area for future research.

#### System design limiting precision and sensitivity

In addition to limitations in separation efficiency and speed, current separation and detection methods lack precision. Most strategies target only one or a few specific marker proteins, which neither provides a quantitative analysis of a specific subpopulation nor allows for a systematic analysis of multiple subpopulations, thus compromising the accuracy of liquid biopsy. Additionally, some of these methods have limited sensitivity, especially when handling small sample volumes, which are common in clinical liquid biopsies.

To address these challenges, we have raised potential future research directions in the field of liquid biopsy: (1) Developing new microfluidic chips that cover more EV subpopulation markers, thereby improving throughput and the accuracy of clinical diagnosis. Alternatively, by designing more complex logical systems, achieve refined separation of EV subpopulations carrying multiple bioactive molecules of the same or different types; (2) Some biological detection technologies such as Proximity Extension Assay (PEA) and Molecular On-bead Signal Amplification for Individual Counting (MOSAIC), which utilizes RCA similar to DNA nanoflowers, offer ultra-high sensitivity 118,119. Although these technologies have not been widely used in EV research or have been limited to detecting tetraspanins, applying them to EV subpopulation detection could significantly enhance the precision and sensitivity of clinical liquid biopsy.

### Downstream experimental research: insufficient integrity and biological activity

In recent years, as the biological functions of EV subpopulations have been increasingly uncovered, the demand for EV subpopulations in downstream research has grown. We have observed that the focus of EV subpopulation separation has shifted from purity, yield, and accuracy to the integrity and biological activity of individual EVs. For downstream research, traditional separation methods based on physical properties may damage the EV membrane structure, while directly using antibodies can be difficult to remove, potentially affecting the biological activity of EVs. Current separation methods based on click chemistry/covalent chemistry enable reversible binding between EV subpopulations and separation materials. However, the released EVs still carry carbon chains that may impact their biological function. Although negative separation strategies preserve the integrity and biological activity of EV subpopulations, non-target subpopulations might remain. Aptamers offer the advantage of reversible separation and can be designed to meet various separation requirements, making them ideal for non-destructive separation. However, most EV subpopulation separations currently utilize single aptamers or DNA nanostructures.

For the separation of EV subpopulations used in downstream research, we suggest the following future research directions: (1) Design and compare different DNA nanostructures for their advantages and disadvantages in EV subpopulation separation; (2) By modifying the nucleic acid sequence, length, and other features of aptamers, achieve refined separation of EV subpopulations carrying multiple bioactive molecules of the same or different types; (3) Apply other DNA nanomaterials containing embedded aptamers, such as DNA-based micelles/polymers and DNA hydrogels, to EV subpopulation separation; (4) Some separation methods based on the electrical, acoustic, or fluidic properties of EVs can preserve EV integrity and biological activity but can only separate EVs from other biological components. Adapting these techniques for EV subpopulation separation will be the primary challenge for this strategy.

### Distinguishing analogous EV subpopulations: different ones from the same cell type or similar ones from different cell types

As discussed earlier, EVs exhibit significant heterogeneity, particularly in the diversity of cargo molecules, which complicates both clinical liquid biopsy and downstream studies of EV subpopulations. To minimize or even eliminate the interference caused by cargo molecule heterogeneity in liquid biopsy and related research, a key question arises: how can we distinguish analogous EV subpopulations—those that are similar but originate from different cell types, or those that are different but come from the same cell type?

For EV subpopulations originating from the same cell type, the challenge lies in the fact that the same parent cell markers are expressed, but the subpopulations carry different sub-markers (typically the molecules of interest). To distinguish these subpopulations, it is necessary to target multi-positive markers, including at least one stable parent cell marker and variable sub-markers. For similar EV subpopulations originating from different cell types, the situation is reversed: the subpopulations carry the same sub-markers but have distinct parent cell markers. In this case, distinguishing these subpopulations requires targeting multi-positive markers, including variable parent cell markers and one stable sub-marker. A potential solution to this challenge is a two-step separation method. In the first step, EVs carrying the shared surface marker are separated—specifically, the same parent cell marker for different EV subpopulations from the same cell type, or the same sub-markers for similar EV subpopulations from different cell types. In the second step, further separation is performed to separate the EV subpopulations. Since many surface markers also function as cargo molecules with specific biological roles, the separating agents—such as antibodies or aptamers—should be removed to free up the binding sites of these markers.

However, this strategy relies on surface markers, which may not be effective for distinguishing subpopulations that carry different internal molecules. Therefore, future research should focus on developing labeling methods for internal molecules to enhance the separation of these subpopulations. Moreover, artificial intelligence (AI) and machine learning (ML) are increasingly playing a critical role in the biomedical field. By integrating AI and ML, their logic and algorithms could potentially offer innovative solutions for addressing the complexity of EV heterogeneity and improving the accuracy of EV subpopulation identification and separation.

## Conclusion

This review provides a comprehensive overview of recent advances in the separation and detection of EV subpopulations. As highlighted, the challenge of balancing accuracy, efficiency, and convenience in separation methods—while ensuring high purity and biological activity of EVs—remains significant. Similarly, achieving comprehensive optimization in the speed, accuracy, and ease of recognition for EV subpopulation detection methods continues to be a hurdle. Nevertheless, the development of effective separation and detection techniques for EV subpopulations is essential for enabling precise liquid biopsies and advancing detailed EV research. Currently, no single method for EV subpopulation separation and detection meets all ideal criteria. Therefore, researchers are encouraged to choose the most suitable method based on factors such as sample volume, type, and experimental objectives, or to combine multiple techniques to leverage their respective strengths.

## Figures and Tables

**Table 1:** The advantages, disadvantages, dependent properties and principle of traditional and some burgeoning separation methods for EVs.

Traditional and Some Burgeoning Separation Methods for EVs		Advantages	Disadvantages	Dependent EV Properties and Separation Principle	
**Traditional Separation Methods**	**Differential Ultracentrifugation**	Gold standard, available for large sample volume, suitable for multiple samples, comparatively high purity	Time consuming, special equipment, destructive to biological properties	Biophysical property: density	Sequentially increasing centrifugation speeds
	**Density Gradient Centrifugation (DGC)**	Comparatively high purity, low loss rate	Time consuming, solvent could influence integrity	Biophysical property: density	Creating density gradient with sucrose or iodixanol
	**Size Exclusion Chromatography (SEC)**	Fast, simple procedure, comparatively well-preserved integrity and biological properties	Comparatively low purity	Biophysical property: particle size	Passing chromatographic column with different speed
	**Ultrafiltration (UF)**	Fast, simple procedure, low cost	Comparatively low purity, clogging	Biophysical property: particle size	Filtrating with external pressure
	**Precipitation Methods**	High acquisition rate, integrity and biological activity preserved with low centrifugation speed	Low purity, polymer could still influence biological properties	Biophysical property: solubility	Altering solubility with polymer (e.g., PEG) and centrifugating with low speed
	**Immunoaffinity Methods**	High purity, convenient for subsequent detection	Antibodies are difficult to disengage, biological properties differed	Biochemical property: antigen antibody affinity/affinity chromatography	Specific tetraspanins (e.g., CD9, CD63, CD81), immune-capture
	**Commercial Kits**	Simple, no special equipment	Varied	Varied	Varied
**Burgeoning Separation Methods**	**Classical Microfluidic Methods**	High integrity, well-preserved biological properties, efficient, comparatively high purity, convenient	Low sample volume, not applicable to subpopulation separation	Biophysical properties: electrical, acoustic, fluidic properties	Controlling direction through external electric, acoustic or fluidic fields
	**Classical Covalent Chemistry Methods**	Reversible capture, comparatively high integrity and well-preserved biological properties	Low sample volume, not applicable to subpopulation separation, residual chemical carbon chain may still influence biological properties	Biochemical properties: chemical properties	Covalent chemical reactions

**Table 2:** The advantages, disadvantages and applications of separation methods for EV subpopulations.

Separation Methods for EV Subpopulations		Advantages	Disadvantages	Applications
**Microfluidic Chip-Based Method**		Rapid, automatic, compatible with multiple indicators	Low throughput, unsuitable for large-scale sample	Small volume, liquid biopsy
**Click Chemistry/Covalent Chemistry-Based Method**		Reversible, relatively high integrity and biological activity	Complex procedure, biological properties slightly altered	Downstream research
**Aptamer-Based Method**	**Multivalent DNA Nanoflowers**	High efficiency, high affinity, traceless release	Complex procedure, limited accuracy	Downstream research, liquid biopsy
	**Robust Separation of Double-Positive EVs**	Multiple indicators, traceless release	Low concentration	Fine liquid biopsy and downstream research
**Receptor-Ligand Interaction-Based Method**		Novel, simple	Low specificity and limited generality	Specific protein separation
**Negative Separation Strategy-Based Method**		Novel, theoretically undamaged, high biological activity	Relatively low purity	Downstream research and liquid biopsy

**Table 3:** The advantages, disadvantages and applications of detection methods for EV subpopulations.

Detection Methods for EV Subpopulations		Advantages	Disadvantages	Applications
**Nanoparticle Flow Cytometry (NanoFCM)**		High throughput, high sensitivity, rapid, qualitative analysis	Limited accuracy for EVs smaller than 100 nm, some with limited resolution	Available for relatively big EVs
**Color/ Fluorescence Reaction-Based Optical Detection Methods**	**Single Molecule Array (Simoa)**	High throughput, rapid, high sensitivity, convenient, naked eye analysis, some automatic	Destructive to EVs for downstream research	Available for small sample volume
	**Horseradish Peroxidase (HPR)-Mediated Color/ Fluorescence Reaction**			
**Ion Concentration Changes-Based Electrochemical Sensor Detection Methods**		High sensitivity, high accuracy, wide detection range, sensitive	Time consuming, destructive to EVs for downstream research	Available for small sample volume and low EV concentration
**Raman Beads**		Enhanced signal, resistant to interference from bioactive substances	Destructive to EVs biological functions	Multi-component sample

## Abbreviations

Ab: antibody
ACE2: angiotensin converting enzyme 2
AI: artificial intelligence
DA: dopamine
DFs: DNA flowers
DGC: density gradient centrifugation
DTT: 1, 4-dithiothreitol
EGFR: epidermal growth factor receptor
ELISA: enzyme-linked immunosorbent assay
EpCAM: epithelial cell adhesion molecule
ES: Ewing sarcoma
EVs: extracellular vesicles
FAM134B: family with sequence similarity 134 member B
HER2: human epidermal growth factor receptor 2
HRP: horseradish peroxidase
IEDDA: inverse electron demand Diels-Alder
LINGO-1: leucine-rich repeat and immunoglobulin-like domain-containing protein 1
Mag-Anti-CD63: magnetic nanoparticles linked to anti-CD63 antibody
miRNA: microRNA
ML: machine learning
MOSAIC: molecular on-bead signal amplification for individual counting
NanoFCM: nanoparticle flow cytometry
PDA: polydopamine
PD-L1: programmed death ligand 1
PEA: proximity extension assay
PEG: polyethylene glycol
QD: quantum dot
RBD: receptor binding domain
RCA: rolling circle amplification
RGP: resorufin β-d-galactopyranoside
SEC: size exclusion chromatography
SERS: surface-enhanced Raman scattering
sEVs: small EVs
Simoa: single molecule array
SiNPs: silica nanoparticles
SiNWS: silicon nanowire substrate
SβG: streptavidin-β-galactosidase
TCO: trans-cyclooctene
T-EVs: tumor tissue-derived extracellular vesicles
Tz: tetrazine
UF: ultrafiltration
3D: three-dimensional
